# Development of very large electrode arrays for epiretinal stimulation (VLARS)

**DOI:** 10.1186/1475-925X-13-11

**Published:** 2014-02-06

**Authors:** Florian Waschkowski, Stephan Hesse, Anne Christine Rieck, Tibor Lohmann, Claudia Brockmann, Thomas Laube, Norbert Bornfeld, Gabriele Thumann, Peter Walter, Wilfried Mokwa, Sandra Johnen, Gernot Roessler

**Affiliations:** 1 Institute for Materials in Electrical Engineering I, RWTH Aachen University, Sommerfeldstr 24, 52074 Aachen, Germany; 2 Department of Ophthalmology, University Hospital Aachen, RWTH Aachen University, Pauwelsstr 30, 52074 Aachen, Germany; 3 Department of Ophthalmology, University Hospital Essen, Hufelandstr 55, 45147 Essen, Germany; 4 Hôpitaux universitaires de Genève, Service d’ophtalmologie, Rue Alcide-Jentzer 22, CH-1211 Genève 14, Suisse

**Keywords:** Retinal prosthesis, Artificial vision, Retinitis pigmentosa, Blindness, Rehabilitation, Vitreoretinal surgery, Silicon wafer Technology, Polyimide, Neurostimulation

## Abstract

**Background:**

Retinal implants have been developed to treat blindness causing retinal degenerations such as Retinitis pigmentosa (RP). The retinal stimulators are covering only a small portion of the retina usually in its center. To restore not only central vision but also a useful visual field retinal stimulators need to cover a larger area of the retina. However, large area retinal stimulators are much more difficult to implant into an eye. Some basic questions concerning this challenge should be answered in a series of experiments.

**Methods:**

Large area retinal stimulators were fabricated as flexible multielectrode arrays (MEAs) using silicon technology with polyimide as the basic material for the substrate. Electrodes were made of gold covered with reactively sputtered iridium oxide. Several prototype designs were considered and implanted into enucleated porcine eyes. The prototype MEAs were also used as recording devices.

**Results:**

Large area retinal stimulator MEAs were fabricated with a diameter of 12 mm covering a visual angle of 37.6° in a normal sighted human eye. The structures were flexible enough to be implanted in a folded state through an insertion nozzle. The implants could be positioned onto the retinal surface and fixated here using a retinal tack. Recording of spontaneous activity of retinal neurons was possible in vitro using these devices.

**Conclusions:**

Large flexible MEAs covering a wider area of the retina as current devices could be fabricated using silicon technology with polyimide as a base material. Principal surgical techniques were established to insert such large devices into an eye and the devices could also be used for recording of retinal neural activity.

## Background

Retinal degenerations such as Retinitis pigmentosa (RP) may cause bilateral blindness. It is estimated that in Germany about 15,000 subjects are legally blind due to this disease. Although it is known that the disease is caused by a variety of mutations in several genes coding for key enzymes involved in retinal metabolism and light processing [1]–[3] no treatment is really established. Since it has been demonstrated that blind RP subjects were able to detect light spots and patterns by electrical stimulation of the retina [4]–[6] visual prostheses have been fabricated, approved, and used in RP patients [7]–[10]. Those implants usually consist of a multielectrode array (MEA) placed underneath the retina or onto the retinal surface and a system for signal and energy transfer [11]–[13]. Up to now stimulating electrode arrays were designed as very small devices for stimulation of the fovea and the perifoveal region (Table 1). However, small devices are not capable to restore a large visual field, because they simply contact only a small area of the retina, e.g. 5 × 5 mm as for the ARGUS II device representing a visual angle of about 18° (or 9° visual field respectively) in a normal sized eye. For blind RP patients the re-activation of a large visual field is important to regain mobility and orientation. Therefore we conducted experiments in fabrication and handling to evaluate whether it is possible to build stimulators for large retinal areas. The challenge was that such large structures must have the flexibility to be inserted through a small incision into the eye without damage of the delicate electronic structures or the eye itself.

**Table 1:** Overview on known sizes (width/height/thickness) of electrode arrays used in retina implants with direct retinal contact and their respective number of electrodes

**Implant**	**Concept**	**# of electrodes**	**Dimensions (w/h/t in mm)**
Retinal implant AG	Subretinal	1500	3/3/0.07*)
Argus II second sight	Epiretinal	60	5/5/0.5**)
EPIRET III	Epiretinal	25	2/3/0.02 [8]

*)E. Zrenner, et al. Visual Sensations Mediated By Subretinal Microelectrode Arrays Implanted into Blind Retinitis Pigmentosa Patients. *Proc. 13th Annu. Conf. of the IFESS*, Freiburg, 2008.

**)V. Kandagor, et al. In situ Characterization of Stimulating Microelectrode Arrays: Study of an Idealized Structure Based on Argus II Retinal implants. *Implantable Neural Prostheses,* Springer New York; 2010. pp. 139-56.

## Methods

### Simulation of material characteristics

To get an idea of the behavior of a polyimide foil within an eye, we created a very simple model for Comsol, a common finite element simulation software. The model consists of an incompressible spherical shell made of steel with an inner diameter of 22 mm and a polyimide foil with a thickness of 100 μm, a disc shape and a circular hole in the center of the structure. Then the foil was vertically moved into the shell until both parts came into contact. The Young’s modulus of the polyimide PI2611 we used in this simulation was 8.5 GPa and was taken from the material data sheet, the poisson ratio of 0.23 was taken from Patel et al. [14]. The friction between the polyimide foil and the shell was disregarded.

### Fabrication process

**Figure 1:**
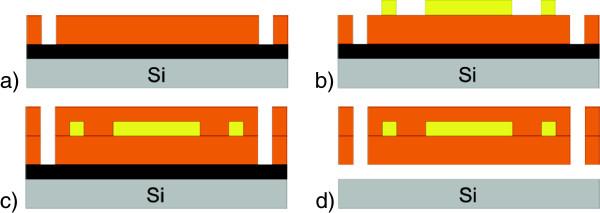
**Fabrication process of VLARS prototypes. a)** 5 μm of polyimide spincoated and structured on Si-Wafer with sacrificial Al-layer (black), **b)** 2.5 μm of electroplated gold for wiring, **c)** another 5 μm of polyimide for isolation and protection of the wiring, **d)** after opening the second PI layer the electrodes are formed by another electroplating step. Further steps include: iridium oxide is sputtered and structured with a lift-off process, and the surface is coated with Parylene C, the electrodes are opened by a dry-etching process with oxygen plasma. Afterwards the devices are separated by etching the sacrificial Al-layer.

Fabrication of the stimulator prototypes takes place on silicon wafers as described in detail in Figure 1. First, wafers are metalized with an aluminium sacrificial layer (1 μm) and with titanium adhesion layers underneath (50 nm) and on top (150 nm). Then a first layer of polyimide PI2611 is deposited by spincoating with a thickness of 5 μm. It is structured by a wet etching process and baked at 400°C under nitrogen atmosphere for 3 hours to polymerize. Now the conducting paths, the electrodes and the contact pads are built by electroplating of gold onto the polyimide with a thickness of 2.5 μm. A chromium layer of 30 nm thickness serves as an adhesion layer. For non-electrically functional test devices we created a fake-wiring to get similar mechanical characteristics as the final electrically functional stimulators and to improve the visibility of the structures during surgery. We also tried to find the optimal positioning of the electrodes. During the next step a second polyimide layer (5 μm) is spincoated on top of the wiring to encapsulate the gold layer. For the second generation of dummy structures we added a Parylene C layer, which will be used in the final devices for additional isolation and protection against moisture. It is deposited onto the devices by a CVD process with a final thickness of 3 μm and patterned by a dry-etching process with oxygen plasma. The thicknesses of all layers were chosen as in the fabrication of the EPI-RET III implant [15]. The electrodes of the final devices are formed by a second electroplating step after structuring the second PI-layer and are coated with reactively sputtered iridium oxide films with a thickness of 500 nm. That leads, due to its high charge delivery capacity, to low electrode impedance which allows the use of low stimulation voltages [15]. Low voltages could increase the durability of the electrodes during long term stimulation experiments. The resulting devices were labeled as VLARS MEA (very large area retinal stimulator - multielectrode array).

### Simulation of the surgical approach

The surgical approach was simulated by performing the procedure in enucleated porcine eyes. The setting was the same as in real surgery. Operations were performed by experienced retinal surgeons using a surgical microscope. Removal of the lens was done by phacoemulsification and removal of the vitreous by vitrectomy using a 20 gauge 3 port approach. Visualization was achieved with endoillumination and a 130° contact lens placed on the corneal surface. Insertion of the device was done either by a scleral incision or by corneal incisions of 6 mm. Insertion of the device was facilitated by using a plastic nozzle.

### *In Vitro* test on stimulation and recording capabilities

Healthy Wistar rats were dark adapted for one hour and decapitated in deep anaesthesia. Retinas were taken from the eye under dim red light. The electrodes of the VLARS MEA were connected to a multichannel recording and stimulating device (MEA2100-System, Multi Channel Systems GmbH, Reutlingen, Germany). The isolated retina was placed onto the VLARS MEA with the ganglion cell side adjacent to the electrodes. The retina was permanently superfused for at least 45 minutes with Carbogene (95% O2; 5% CO2) saturated Ames Medium (Sigma-Aldrich, St. Louis, MO, USA). For electrical stimulation a biphasic 40 μA current pulse with a cathodal and anodal phase duration of 500 μs was chosen. Light stimulation was done using a 1 second white light flash. The stimulation and recording was done with the MEA-Software MC_Rack (Multi Channel Systems GmbH, Reutlingen, Germany). For spike detection raw data sets of 3-5 minutes continuous recording were analyzed with a 300 Hz high-pass filter and a 3000 Hz low-pass filter and an amplification gain factor of two. The data sampling rate was 25 kHz/channel. To distinguish spike waveforms on one electrode spikes were sorted computer based and manually with spike sorting software Offline SorterTM (Plexon Inc, Dallas, Tx, USA) before response analysis was done (NeuroExplorerTM, Nex Technologies, Madison, AL, USA).

All animal experiments were done in accordance with the ARVO statement for the use of animals in ophthalmic and visual research [http://www.arvo.org/About_ARVO/Policies/Statement_for_the_Use_of_Animals_in_Ophthalmic_and_Visual_Research/] and in accordance to the German Law for Animal Protection after approval obtained from the local government.

## Results

### Concept and assumptions

The VLARS stimulation array is based on the fabrication process of the EPI-RET III implant, which uses the epiretinal concept, i.e. the electrode array is placed on the inner surface of the retina [16]. During implantation the device must be inserted into the eyeball through a small corneal incision. After removal of the lens and of the vitreous body the device can be pushed through the anterior chamber into the vitreous chamber of the eye. From this position it can be further moved to the retinal surface where it can be placed onto the posterior pole. Stable fixation is provided with a retinal tack. We designed a VLARS stimulator with a diameter of 12 mm covering an area of about 110 mm^2^. A retinal stimulator of that size covers a visual angle of 37.6° in an eye of normal size (axial length 22.8 mm) representing a visual field of 18.8°. With our technology, more than 250 electrodes can be realized on such a base structure for a larger field of percepts and to improve the spatial resolution in the center of the device compared to current available epiretinal technology.

### Design

**Figure 2:**
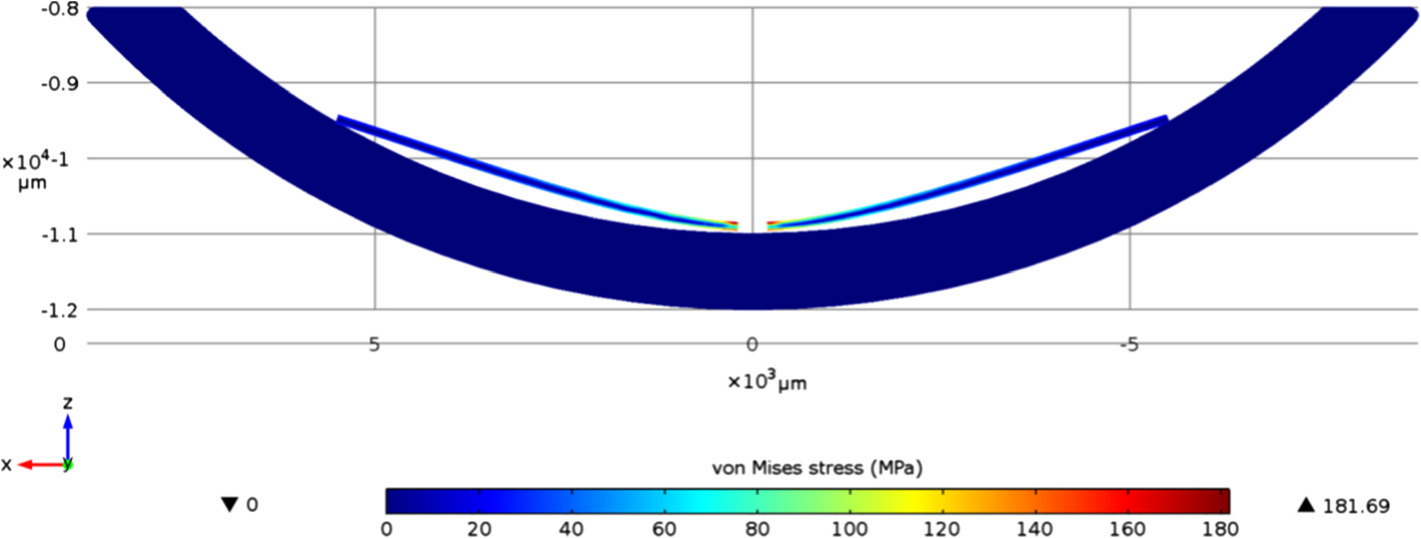
**Simulation of the behaviour of a polyimide foil placed in a spherical shell.** Displayed is a sectional drawing of the model with the von Mises stress of the PI-foil in MPa. Schreibweise: behavior/behavior (s. Text)

Shapes for the flexible base structure of the electrode array were identified which can be easily bent without damage. A foldable design is required to avoid large incisions for implantation during surgery. This may reduce the corneal incision size down to 5-6 millimeters. The second critical design parameter is the ability of the structure to fit to the three-dimensional curved shape of the retina. Because the implant is fabricated on the wafer level with planar processes it has a completely flat profile, while the retina is curved. For successful stimulation the device needs to be stretched or compressed to bring the electrodes in contact with the retina over the whole area. In Figure 2 results of a numerical simulation using FEM on the mechanical behavior of a polyimide foil placed in a curved shell are shown. In the cross-section it is obvious and expected that parts of the implant are not in contact with the retina due to absence of stress-relieving patterns. At the centre the foil is pressed down and in full contact with the ground as well as at the outer edges. In between, the structure does not adapt to the curvature and eventually lifts up from the tissue. These issues can be addressed in two ways. The first method would be to create concentric segments within the structure. This would however make the handling of the structure more difficult during implantation. The other possibility is to create the devices with an inherent curvature. This could be done by utilizing the thermal and mechanical properties of the Parylene coating. As our primary concern is the handling of the stimulator during implantation we chose the second method.

### Material selection

We integrated the MEA into the polyimide PI2611 (HD MicrosystemsTM GmbH, Neu-Isenburg, Germany) foil. The advantage of this material is its relatively low moisture uptake of 0.5%, which helps to prolong the durability of the system embedded metallization layers in the prevailing wet conditions. Furthermore polyimide is characterized by a low relative dielectric constant of about 2.9, which also provides an excellent electric isolation of the feed lines of the electrodes. The low intrinsic stress of PI2611 makes it suitable as a base material for stimulators as the intended one. Additionally, polyimide can be easily structured via photolithographic techniques as it is soluble in the developer of standard photoresists. The Parylene C encapsulation serves as an additional moisture barrier due to its hydrophobic behavior. Additionally Parylene C is moldable under the influence of elevated temperatures. This opens the possibility of creating curved structures [17].

**Figure 3:**
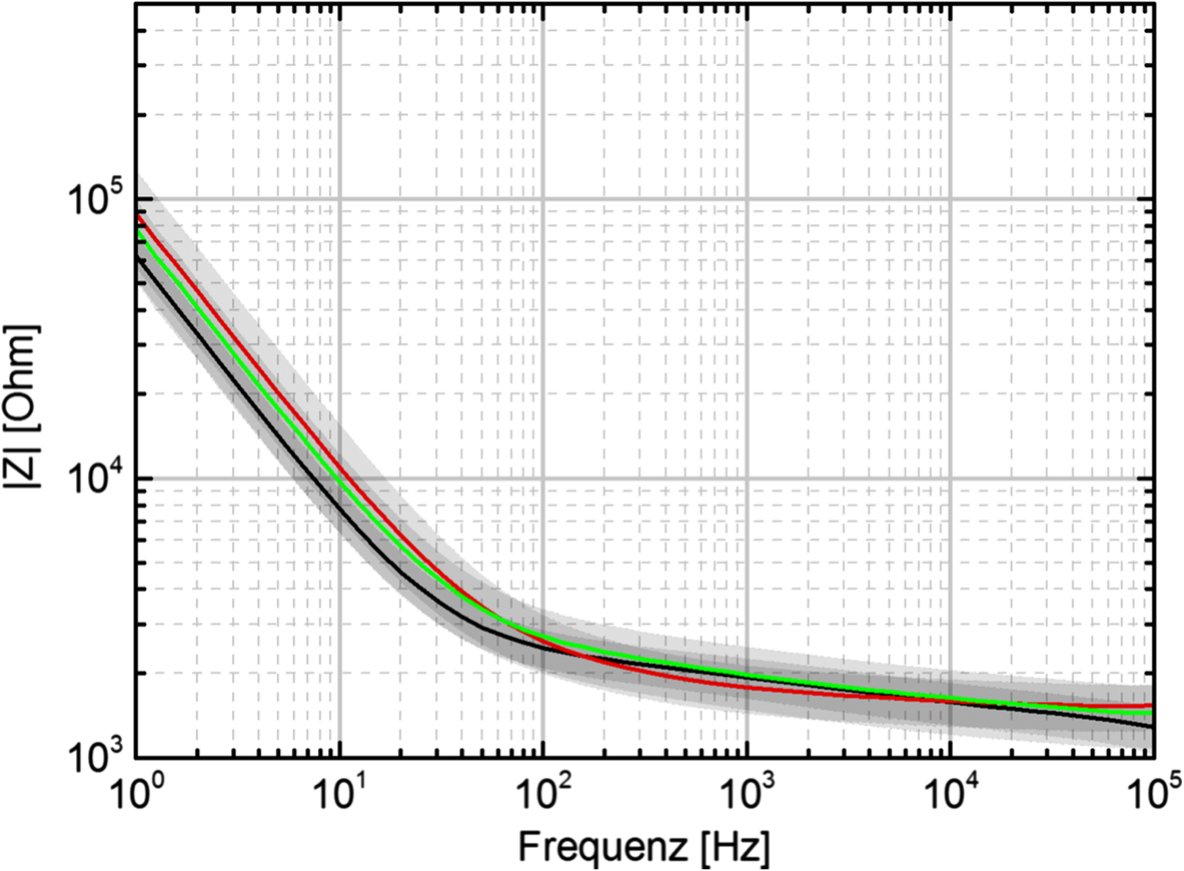
**Impedance spectra from electrodes of three VLARS devices.** Each of the colored lines show the average of all 25 electrodes of one device. The grey areas represent the standard deviation.

**Figure 4:**
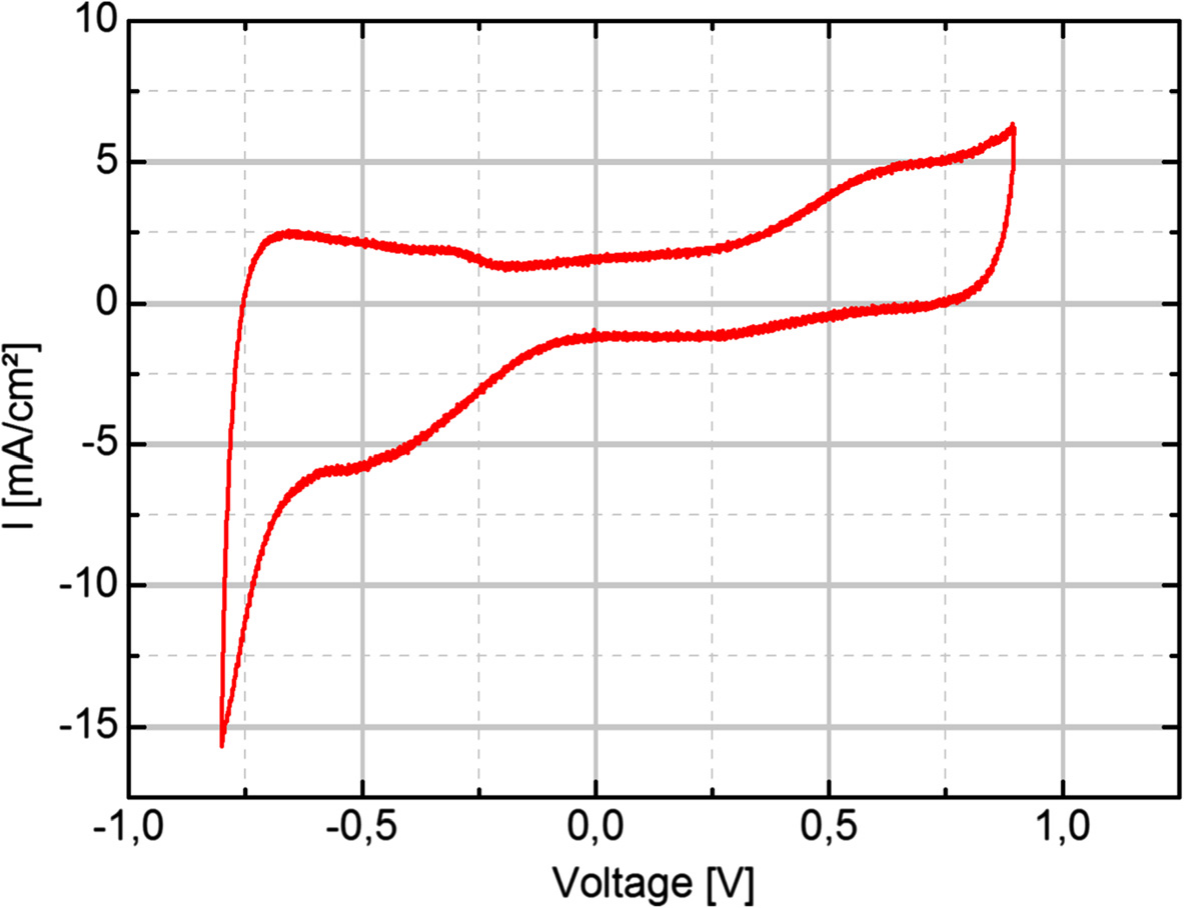
Typical cyclovoltametry measurement of VLARS electrodes.

The 250 electrodes of the final VLARS prototypes are circular shaped with a diameter of 100 μm. 25 of the electrodes were connected to an external plug connector for stimulation and recording purposes. The impedance spectra of the electrodes of three devices are shown in Figure 3. At 1 kHz, a common stimulation frequency, the average impedance varies around 2 kΩ for all devices. The standard deviation is around 500 Ω. Figure 4 shows a cyclovoltametric (CV) measurement of the iridium oxide electrodes. It was carried out in a voltage range of -0.8 V to 0.9 V with a ramp speed of 100 mV/s. The calculated charge delivery capacities of the VLARS electrodes are in the range of 40 to 50 mC/cm^2^, which is consistent with the literature [18].

### Shape selection and design modification

**Figure 5:**
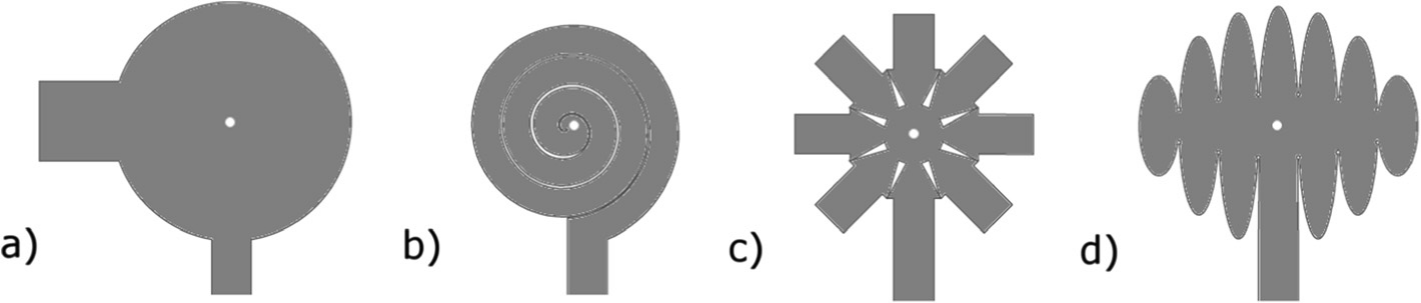
Designs of the first generation of VLARS-prototypes showing the designs D1 (a), D2 (b), D3 (c) and D4 (d).

Figure 5 shows the different designs of the first generation that were tested in enucleated porcine eyes. A continuous, circular shape (D1) served as control. A spiral shape (D2) was constructed because we thought that it could be easily inserted by turning the device arms through a small sclerotomy. Moreover, slits between the spiral windings would have allowed a better fitting to the surface profile of the retina. However, implantation revealed remarkable problems of handling and placing the soft implant onto the retinal surface. Additionally, the structure design included an increased risk of material damage during implantation.

The star-like shape (D3) was designed because of its theoretical very good unfolding potential. During insertion (surgery) the arms were properly (nicely) bent back and regained their original shape inside the eye without damage of the device structure. Also it fits well to the curvature of the eyeball. The globe design (D4) is based on the principle of an interrupted cylindrical projection of a spherical structure on a flat surface.

All first generation designs D1-4 had a central hole for tack fixation. Additional holes were provided in the periphery of the structure to be used if necessary to support the primary fixation and keep the electrodes in contact with the retina.

**Figure 6:**
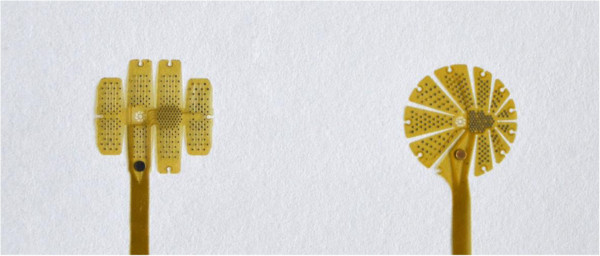
**Last design revisions that are going to be used for the functional devices.** Left, globe design. Right, star design. In both designs electrodes are arranged in two functional zones, a high density area intended to be placed at the fovea and a low density zone for contacting more peripheral parts of the retina.

Following first implantation experiments in porcine eyes the prototypes D3 and D4 were selected for further development. Both designs were modified in a way that the effective area was enlarged by reducing the gaps between the blades and by increasing their number as shown in Figure 6. The symmetry of the patterns is abandoned to provide the highest density of electrodes in the center of the structure for stimulation of the foveal region whereas in the more peripheral areas the electrode density is less corresponding to the lower density of ganglion cells and the lower spatial resolution provided by these retinal areas. The tack fixation site was moved from the center to a distant position opposite to the optic nerve head to reduce the effect of tissue reaction at the tack on the stimulation process.

### Surgical simulation

**Figure 7:**
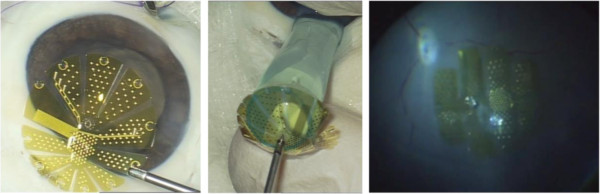
**Left: VLARS MEA with star design before implantation into the pig eye.** Center: Insertion of the VLARS MEA into the implantation nozzle. Right: Globe type MEA placed onto the retinal surface of a pig eye and fixated with a retinal tack.

Implantation tests in enucleated eyes of pigs and rabbits demonstrated that a transscleral approach to insert these devices may cause a phase of severe hypotony even if the eye was filled with perfluorocarbon liquids (PFCLs) and even if a PFCL filled nozzle was used as an insertion aid. Insertion was easier and better controlled if the stimulator was positioned through a corneal incision as seen in Figure 7. It was also evident, that the D3 and D4 designs were much more suitable in terms of smooth folding and unfolding. Both devices could be fixated onto the retinal surface using a single retinal tack (Figure 7, right panel). The surgeon rated the procedure as complex but feasible and safe. This rating does not differ from ratings of implantation of clinically available retinal implants such as the ARGUS II device.

**Figure 8:**
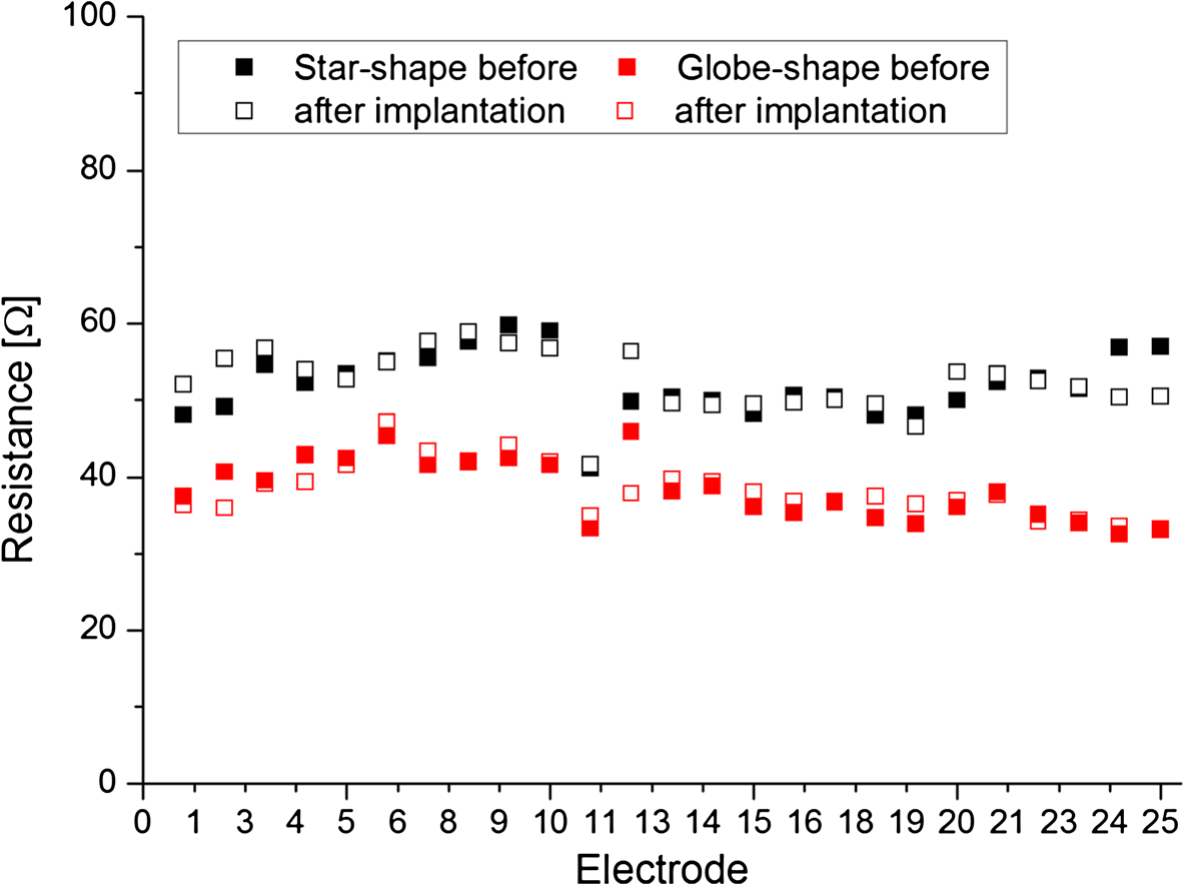
Measurements of the ohmic resistance of the contacted electrodes before and after implantation shows no signs of damage by the implantation experiment.

We measured the ohmic resistance between the contact pads and the respective electrodes before and after an implantation experiment to verify, that no damage was done to the electric wiring. Figure 8 shows no significant change in the ohmic resistance on any of the electrodes.

### Preliminary test on recording capabilities

**Figure 9:**
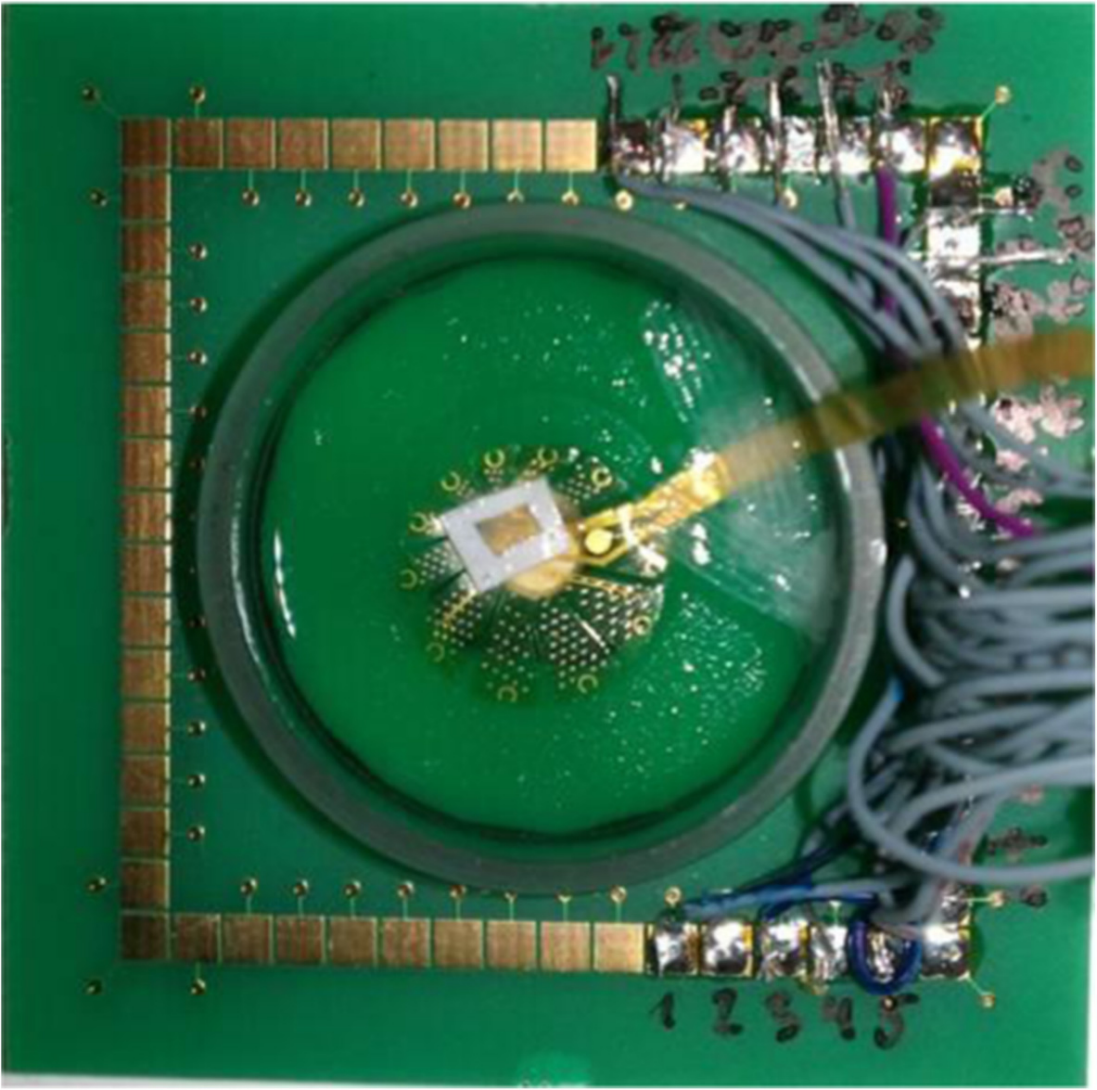
**Functional testing of a star type VLARS MEA.** For recording of spontaneous and evoked spike activity a piece of the retina (arrow) from a Wistar rat was placed with the ganglion cell layer onto the VLARS-MEA. The bond pads on the right are connected to the MEA hardware equipment for recording and stimulation. The wires connect each of 25 active VLARS electrodes with the bond pads.

**Figure 10:**
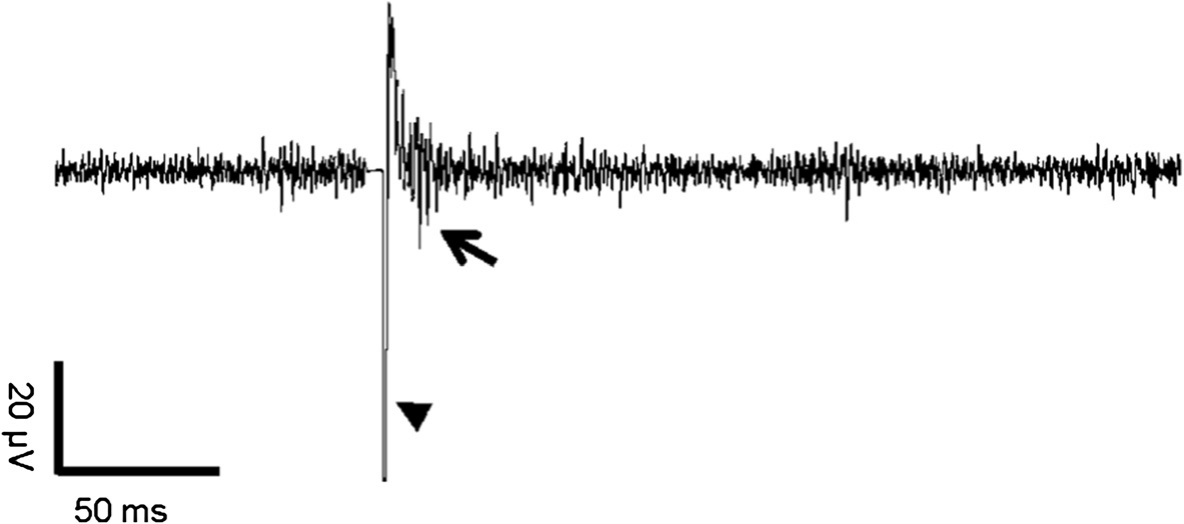
**Monitoring of neural activity after stimulation in the isolated rat retina using a VLARS MEA.** Arrowhead shows the stimulation artifact of the biphasic current pulse. The neural response could be detected as evoked spikes marked with an arrow.

**Figure 11:**
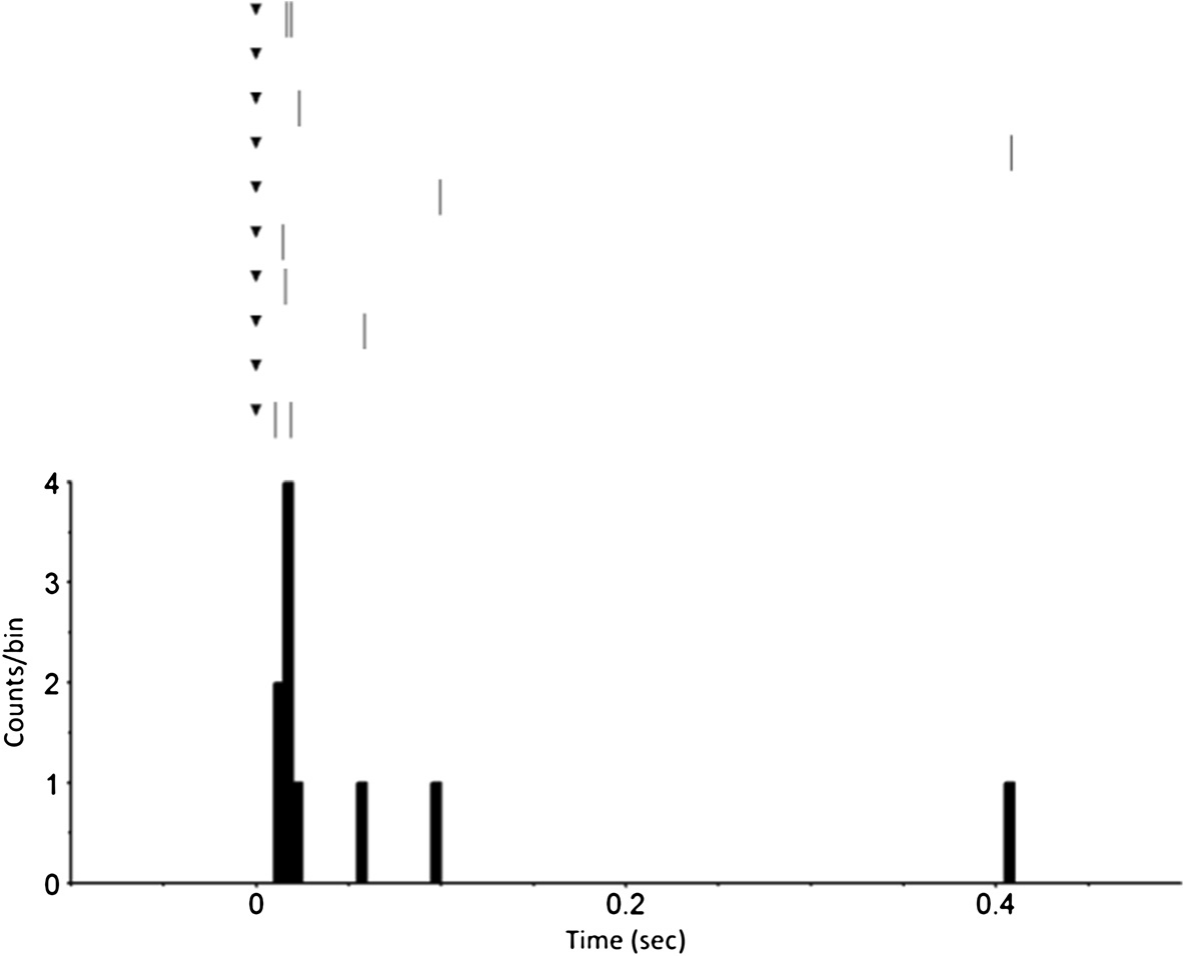
**Perievent raster with histogram of 10 trials.** The response of a wild type Wistar rat retinal ganglion cell to a biphasic current pulse recorded with a VLARS MEA. The pulse started at time point “0” (black triangle). The perievent histrogram (lower trace) summarizes the results of the corresponding perievent raster (upper traces). The neural response could be detected immediately after stimulation. Bin size 5 ms.

The VLARS MEA was connected to the MEA2100-Setup as shown in Figure 9. On the region with the highest electrode density (foveal region), a piece of Wistar wild-type retina was placed with the ganglion cell layer facing the electrodes. For a preliminary assessment of stimulation and recording properties of the VLARS MEA a biphasic current pulse was delivered at one electrode. The stimulation artefact recorded from an adjacent electrode was clearly visible but also a neural response with increased spike activity after the pulse as shown in Figures 10 and 11. In Figure 11 every spike is indicated as a black bar and every row shows one trial. The perievent histogram (Figure 11, lower traces) summarizes the data of the individual stimulation trials.

**Figure 12:**
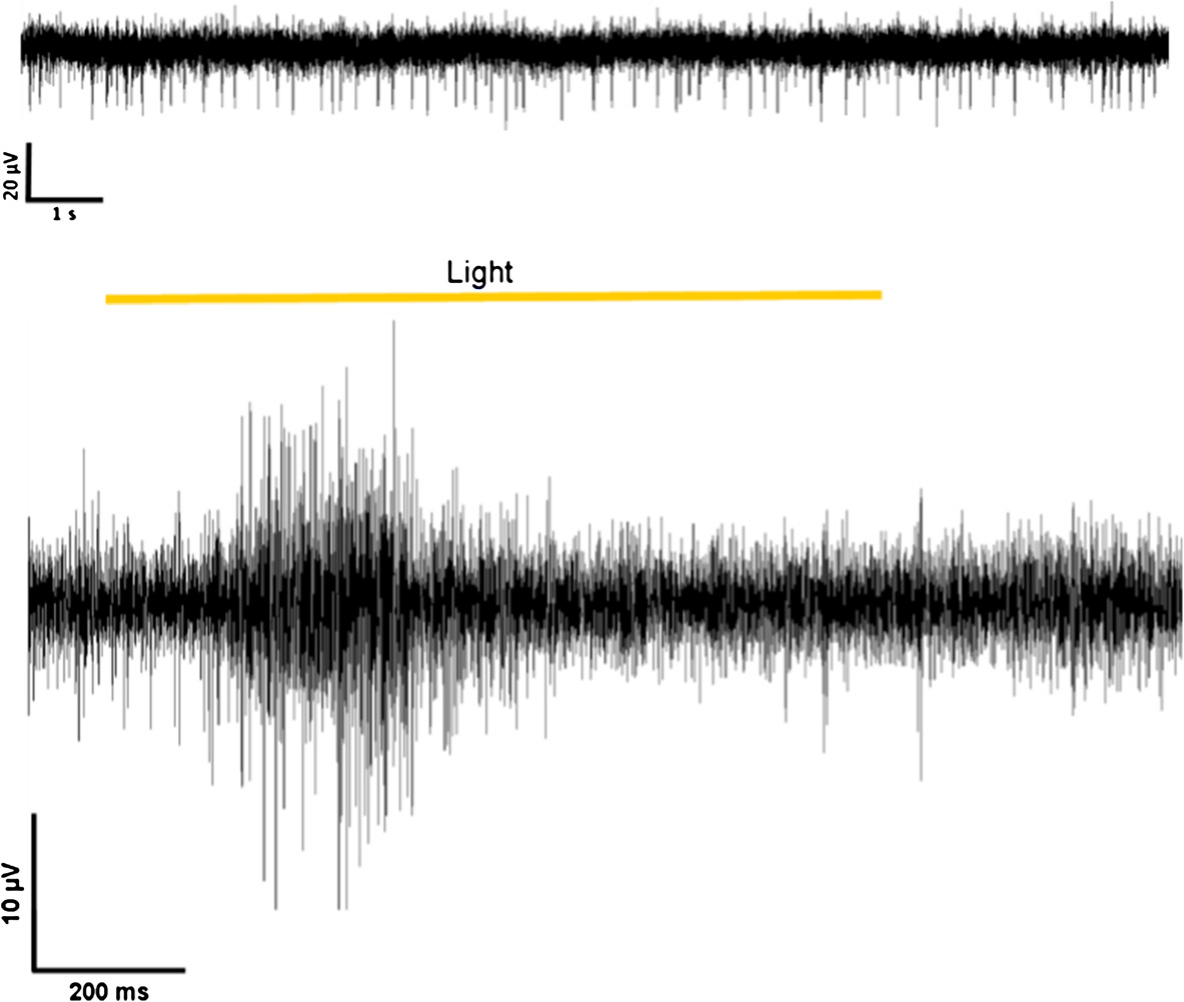
**Monitoring of neural activity in the isolated rat retina using a VLARS MEA.** Top, spontaneous spiking activity of retinal ganglion cells. Bottom, ganglion cell response to light stimulation. Representative recording from one of the connected electrodes.

**Figure 13:**
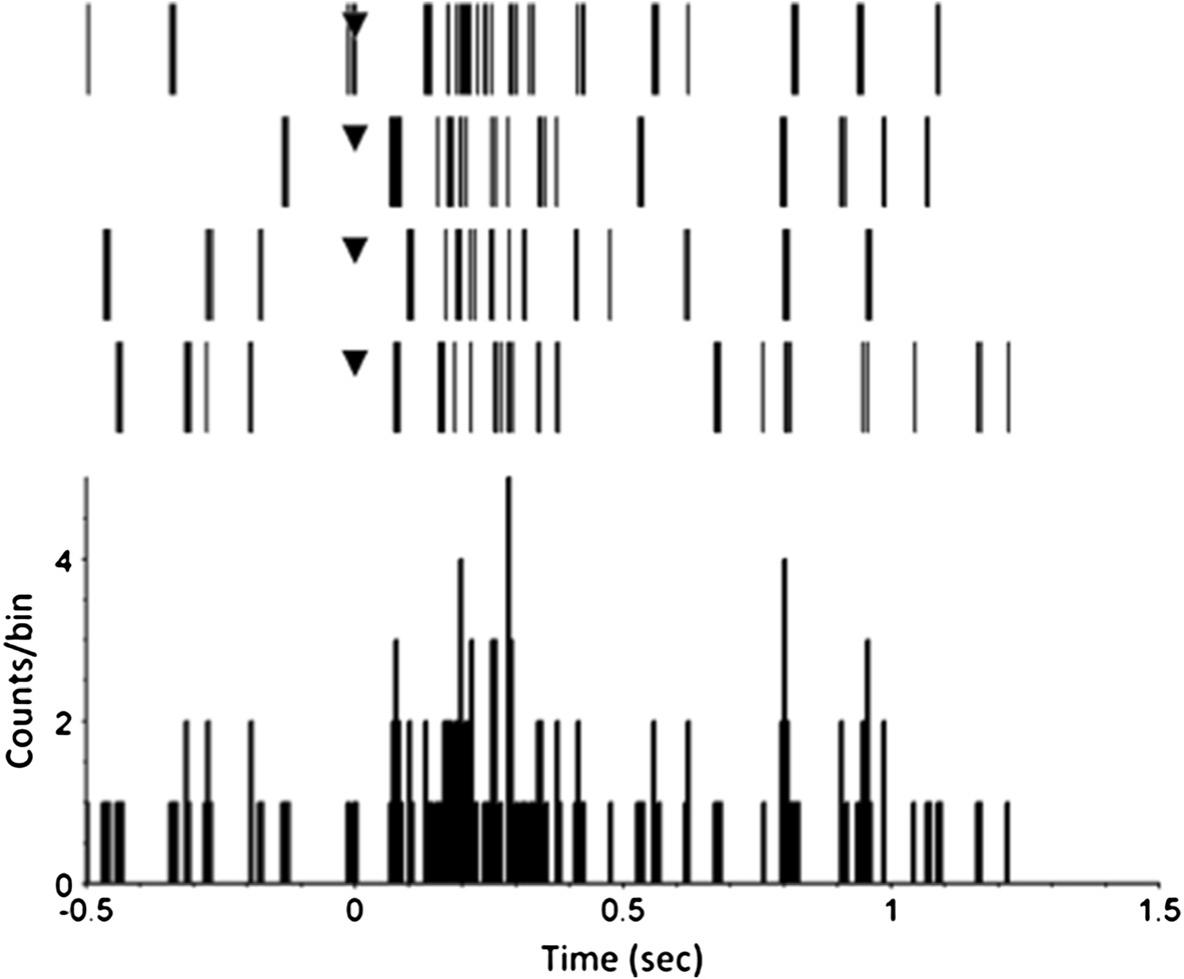
**Perievent raster with histogram of four trials.** The response of a wild type Wistar rat retinal ganglion cell to a one second white light flash recorded with a VLARS MEA. The illumination started at time point “0” (black triangle). The graph shows spontaneous activity 500 ms before stimulus onset, the response during the one second light stimulus and the activity 500 ms after illumination. Bin size 5 ms.

In addition to the studies of the electrical stimulation properties, the recording characteristics of the device were examined in further experiments. Figure 12 (upper trace) demonstrates that spontaneous firing activity of a retinal ganglion cells could be recorded with spikes clearly detectable. In further experiments, the retina was illuminated for 1 second with a white light flash. The response to the light pulse could be monitored at several electrodes as an increased firing activity of the displayed ganglion cell (Figure 12, lower trace). The apparent action potentials were sorted and analyzed. Figure 13 (upper traces) shows the responses to light stimulation of four trials in a perievent raster plot. Every spike is figured as a black bar and every row shows one trial. Illumination started at time-point 0. The time range from -0.5 s to 0 s indicates the spontaneous firing activity of the neuron under red light. The perievent histogram (Figure 13, lower traces) summarizes the data of the individual light trials. After about 80 ms an increase of the spiking activity could be recorded as a response to the light stimulus. With the VLARS MEA it was possible to monitor light responses at seven of the nine connected foveal electrodes.

## Discussion

Stimulators covering a large area of the retinal surface needed to be fitted to the retinal surface topography. The inner surface of the eye is different from an ideal spherical curvature and moreover due to different axial sizes of eyes the inner curvature also differs within individuals. Despite the fact that, individually designed and fabricated stimulators would be great to ensure a perfect fit with the individual surface topography, but the first step in the development of large arrays is the fabrication of a general design which fits for many sizes of eyes as a proof of concept device. Larger variations are considerably increasing the complexity of the fabrication process and are currently not regarded as useful since e.g. very small eyes such as in young children are not considered to be implanted with retinal implants. Implant structures specially designed for animal experiments covering an extreme variety of eye sizes from mouse to pigs were not planned to be fabricated within this project.

In the foveal region the image size on the retina is nearly proportional to the visual angle. An image covering three millimeters in length on the retina corresponds to a visual angle of approximately 10 degrees [19]. Moreover, it has been shown that there is a strong correlation between the position of the stimulating electrode on the retinal surface and the response within the visual cortex and the visual field, respectively [20,21].

Retinal stimulator MEAs can be placed either underneath the retina as subretinal devices or onto the retinal surface as epiretinal devices [7]–[10]. Both approaches have in common that current prototypes are designed for stimulation of the foveal region which may allow a visual angle of about 10° – 18° or 5° – 9° of visual field, respectively.

The VLARS project was started to develop a stimulator MEA covering a larger visual field. We built a device with a diameter of 12 millimeters covering a retinal area of about 110 mm^2^. A retinal stimulator of that size could restore a visual field of about 18.8° covering a corresponding visual angle of 37.6° when implanted in a human eye of normal size. Additionally, more than 250 electrodes can be placed on such a device with current available technology.

Large stimulators were also suggested by Villalobos et al. and the Bionic Vision Australia Group: They described a MEA which can be placed in the suprachoroidal space with dimensions of 8 × 13 mm, providing a visual angle in the human eye of about 27.14 × 39.8° or a visual field of 13.6 × 19.9°. MEA systems not in direct contact with the retina seemed to be limited in terms of spatial resolution because of the distance between the active electrodes and the target neurons. The advantage of suprachoroidal implants however might be the low risk profile of the surgery [22,23].

Studies on the safety of the EPIRET III device, which was also based on polyimide and contained similar materials and the same encapsulation as the VLARS prototypes confirmed a good biocompatibility and function of the implants [24]–[27]. For epiretinal fixation of the device retinal tacks have been shown to provide direct contact of the electrode array to the retinal surface-a prerequisite for successful retinal ganglion cell stimulation (re-detected and re-established) [28]–[30]. While the fixation of small stimulators requires up to two retinal tacks [7,8] it requires further experiments to determine if larger devices will need more elements for fixation. For this reason our next step will be the implantation in animal experiments to prove the biocompatibility of the device and during follow-up examinations to quantify the number of necessary tacks. As optical coherence tomography (OCT) has been successfully used to demonstrate the contact of epiretinal stimulators with the retinal surface [31], this examination procedure will be used for the upcoming experiments. Moreover, stimulating tests will be performed to determine and to evaluate stimulation thresholds of different electrodes in regard to the retinal contact of each part of the stimulator. Alternative approaches to avoid mechanical fixation tools have been described in animal experiments but were not used clinically: Thermosensitive glues and biochemical adhesion provided by immobilized peptide chains have been used in experimental settings [32,33]. In the future these approaches may help to reduce the amount of retinal tacks for fixation of large epiretinal stimulators.

The main concern for large stimulators is the possibility of intra- or postoperative complications. The larger incision necessary to implant such devices into an eye could cause a higher risk of infection, high astigmatism, leakage, and proliferative tissue reactions. Therefore, a flexible device is desirable which can be inserted through a smaller incision. Our experiments on enucleated pig and rabbit eyes showed that when using a nozzle the folded device could be inserted through an incision of 5-6 mm, which seems to be reasonable in terms of risk profile. Stimulation tests and impedance measurements on implanted devices are necessary to demonstrate that stimulators endure the implantation without any damage.

Currently available epiretinal implants have certain drawbacks not only concerning their stimulator size but also concerning electrode size and material, and stimulation algorithms. To yield better spatial resolution a higher density of electrodes is desirable although that usually means to design smaller electrodes with a greater risk of adverse electrochemical reactions at their surface. Such electrochemical reactions have to be avoided by proper selection of the electrode surface material. Stimulation algorithms usually do not take into account that not only ganglion cells may be stimulated but also other excitable cells like amacrine cells or even bipolar cells [34]. The activation of possibly inhibiting local neural networks in the degenerated retina may prevent useful ganglion cell activity. Furthermore, high current or voltage stimulation could evoke action potentials in axons whose somatas are far away from the stimulation site. This may induce percepts coming from a retinal region unrelated to the position of the electrode [35,36]. These factors have to be taken also into account when future implant concepts are discussed. However, the enlargement of the stimulator area may be one step towards a larger visual field in otherwise blind subjects.

We were able to demonstrate in a preliminary experiment, that the device is useful for electrical stimulation of the retina and that it also has the potential to record neural network activity. The latter allows the realization of bidirectional implants capable to record from the target tissue and to stimulate the tissue. The recordings could probably be used to automatically modify and optimize the stimulation pattern, which is usually based on image characteristics and fixed assumptions on the functional state of the retina.

## Conclusions

The successful fabrication of VLARS prototypes and the promising observations concerning surgical handling, stimulation and recording represent first steps in the realization of a large stimulating array for wide-field direct epiretinal stimulation to generate artificial vision with enlarged visual fields. Further experiments are necessary to demonstrate its long-term biocompatibility in-vivo and also its stimulation properties.

## Competing interests

The authors declare that they have no competing interests.

## Authors’ contributions

FW performed the CAD design of the prototypes and simulated the mechanical properties of the VLARS MEAs and fabricated the prototypes under the supervision of WM. He wrote significant parts of the manuscript. SH did the recording and stimulation experiments with the VLARS MEAs and wrote the specific parts of the manuscript. ACR and TL prepared the surgical experiments and were responsible for the documentation. They did the literature search. CB and TLaube assisted in the surgical experiments and contributed extensively to the design concepts discussion and handling evaluation. NB, WM, GT, SJ, and PW developed the concept of VLARS MEAs, wrote the grant application, and wrote significant parts of the manuscript. They finally approved the paper. GR and GT performed the surgical handling tests and wrote the corresponding sections of the manuscript. All authors read and approved the final manuscript.
